# Health Consequences of Sarcopenic Obesity: A Narrative Review

**DOI:** 10.3389/fendo.2020.00332

**Published:** 2020-05-21

**Authors:** Eun Roh, Kyung Mook Choi

**Affiliations:** Division of Endocrinology and Metabolism, Department of Internal Medicine, Korea University College of Medicine, Seoul, South Korea

**Keywords:** aging, sarcopenia, obesity, lean body mass, muscle strength, morbidity

## Abstract

Sarcopenia is defined as the age-related loss of muscle mass and strength or physical performance. Increased amounts of adipose tissue often accompany sarcopenia, a condition referred to as sarcopenic obesity. The prevalence of sarcopenic obesity among adults is rapidly increasing worldwide. However, the lack of a universal definition of sarcopenia limits comparisons between studies. Sarcopenia and obesity have similar pathophysiologic factors, including lifestyle behaviors, hormones, and immunological factors, all of which may synergistically affect the risk of developing a series of adverse health issues. Increasing evidence has shown that sarcopenic obesity is associated with accelerated functional decline and increased risks of cardiometabolic diseases and mortality. Therefore, the identification of sarcopenic obesity may be critical for clinicians in aging societies. In this review, we discuss the effect of sarcopenic obesity on multiple health outcomes and its role as a predictor of these outcomes based on the components of sarcopenia, including muscle mass, muscle strength, and physical performance.

## Introduction

Sarcopenia is a condition characterized by the loss of muscle mass and strength or physical function that naturally occurs with aging. Cachexia is another cause of loss of muscle mass that occurs in diseases such as cancer or immunodeficiency diseases. Baumgartner was the first to propose the term sarcopenic obesity (1), defined as sarcopenia accompanied by an increase in the amount of adipose tissue. A confluence of two epidemics affects this condition: namely, an aging population and an increasing rate of obesity. Sarcopenia and obesity share common pathophysiologic mechanisms, including lifestyle behaviors, hormones, and immunological factors, all of which may act synergistically to affect the risk of developing a series of adverse health consequences. A longitudinal study reported that visceral obesity resulted in a loss of skeletal muscle mass (2). Thus, obesity and sarcopenia may act synergistically, and sarcopenic obesity may have a greater effect on metabolic disorders, cardiovascular disease (CVD), and mortality than either obesity or sarcopenia alone. In this review, we assessed the health consequences of sarcopenic obesity, particularly in age- and obesity-related metabolic diseases. Due to heterogeneity in the definitions and classifications of sarcopenic obesity, we also discuss the role of sarcopenic obesity as a predictor of multiple health outcomes according to each component of sarcopenia, including muscle mass, muscle strength, and physical performance.

## Definition of Sarcopenic Obesity

The current definitions of sarcopenic obesity are based on the individual definitions of sarcopenia and obesity. However, these definitions vary considerably, causing difficulties in making an accurate diagnosis, performing epidemiologic studies, and developing treatment strategies for this disease. Our previous study assessed the ratio of visceral fat to thigh muscle area, which is associated with metabolic syndrome, as a single indicator reflecting sarcopenic obesity (3).

### Definition of Sarcopenia

There remains no consensus regarding the appropriate cutoff points for sarcopenia. Baumgartner et al. first defined sarcopenia as an appendicular lean muscle mass (ALM) divided by height^2^ less than two standard deviations (SDs) below the mean of a young reference group, as measured via dual X-ray absorptiometry (DXA) (4). Janssen et al. used skeletal muscle mass index (SMI = skeletal muscle mass/body mass × 100) measured via bioimpedance analysis (BIA) to define sarcopenia in older Americans (5). Newman et al. evaluated two definitions of sarcopenia in the Health, Aging and Body Composition (Health ABC) Study, which were as follows: ALM divided by height^2^ and ALM divided by height and fat mass (6). They reported that the definition proposed by Baumgartner (ALM divided by height^2^) was strongly correlated to body mass index (BMI) and could therefore identify few individuals with sarcopenic obesity. In 2006, Newman et al. showed that muscle strength has a more important role than muscle mass in assessing mortality (7). Moreover, they reported that handgrip strength can provide risk estimates like those provided by quadriceps strength. The concept of dynapenia, a condition characterized by a decline in muscle strength with aging, was proposed in 2008 (8), and its significance in assessing muscle strength was subsequently highlighted (9, 10).

In 2010, the European Working Group for the Study of Sarcopenia (EWGSOP) (11) required the presence of both low muscle mass and low muscle function (strength or performance) for the diagnosis of sarcopenia. They recognized that muscle strength is not based solely on muscle mass and that the relationship between strength and mass is bidirectional rather than linear (12, 13). Moreover, older adults show a non-linear relationship between leg strength and gait speed; these results provide a mechanism by which small changes in muscle strength produce large effects on physical performance (as assessed by gait speed) in frail adults (14). Thus, the EWGSOP has developed a suggested algorithm for sarcopenia screening using a gait speed of <0.8 m/s before the measurement of muscle mass or strength. The International Working Group for the Study of Sarcopenia provided a consensus definition for sarcopenia: namely, the combination of low appendicular lean mass and poor physical functioning (gait speed <1 m/s) (15). Moreover, they have indicated that patients with a habitual gait speed <1.0 m/s should be considered for the quantitative measurement of body composition using DXA. The Foundation for the National Institutes of Health (FNIH) Sarcopenia Project (16) reported an indirect but causal relationship between muscle mass and function based on their definition of sarcopenia. The FNIH recommended the assessment of low lean muscle mass (ALM: <19.75 kg for men and <15.02 kg for women) using DXA and reduced muscle function (<26 kg for men and <16 kg for women) by handgrip, with adjustment for sex-specific cutoff points according to BMI (ALM/BMI cutoff values: <0.789 for men and <0.512 for women; grip strength/BMI cutoff values: <1.0 for men and <0.56 for women). Moreover, the organization consciously avoided the use of the term sarcopenia to differentiate qualitative (strength) and quantitative (mass) components and showed that individuals with poor physical function require different interventions according to the criteria of sarcopenia (low mass or low strength). In our study, appendicular skeletal muscle (ASM)/BMI-defined sarcopenia was more closely related to cardiometabolic risk factors than was ASM/height^2^ or thigh muscle cross-sectional area (tmCSA)/weight-defined sarcopenia (17). The Asian Working Group for Sarcopenia proposed a diagnostic algorithm based on the evidence currently available in Asia (18). This group followed the EWGSOP approach for the definition of sarcopenia (low muscle mass plus low muscle strength and/or physical performance). However, unlike the method used by the EWGSOP, they included handgrip strength and gait speed in the initial screening. They provided different cutoff points for low muscle mass using DXA (ALM/ht^2^: <7.0 kg/m^2^ in men and <5.4 kg/m^2^ in women) or BIA (ALM/ht^2^: <7.0 kg/m^2^ in men and <5.7 kg/m^2^ in women), low muscle strength using handgrip strength (<26 kg for and <18 kg for women), and low physical performance using gait speed (<0.8 m/s).

In 2018, the updated EWGSOP2 aimed to increase the early detection and treatment of sarcopenia and its risk in clinical practice (19). The group adopted low muscle strength as a principal determinant of sarcopenia because muscle strength is considered better than muscle mass in predicting adverse outcomes (20–22). The EWGSOP2 focused on low muscle strength (grip strength) as the primary parameter of sarcopenia, used low muscle quantity and quality to confirm the sarcopenia diagnosis (DXA or alternatives), and suggested measures of physical performance (gait speed or Timed Up and Go test) to assess the severity of sarcopenia.

### Definition of Obesity

Obesity is a chronic metabolic disease characterized by an increase in body fat stores that consequently elevates the risk of metabolic diseases, CVD, and mortality. As with sarcopenia, a consensus is currently lacking regarding the appropriate cutoff points for obesity. The World Health Organization (WHO) uses BMI to define obesity (≥30 kg/m^2^) and overweight (25–29.9 kg/m^2^) (23). East Asians generally have higher body fat percentages than non-Asians at the same BMI (24). Thus, East Asians have lower BMI cutoff points for obesity (≥25 kg/m^2^) and overweight (23–24.5 kg/m^2^) (25). Although BMI is a useful indicator of body fat in clinical practice, body fat distribution has a better predictive ability than BMI for the development of metabolic syndrome and risk of CVD (26). The American Association of Clinical Endocrinology (27) recommends the use of the WHO body fat thresholds for the diagnosis of obesity (>25% in men and >35% in women). The amount of abdominal fat is easily assessed using waist circumference (WC), which is highly correlated to intra-abdominal fat content. The WHO also used WC thresholds (men: ≥102 cm and women: ≥88 cm) as a surrogate for visceral fat. Lower cutoff points for central obesity are required for different ethnic groups, including Asians (28). The recommended cutoffs are ≥90 and ≥80 cm in Asian men and women, respectively. The Korean Society for the Study of Obesity defines abdominal obesity as WC ≥90 and ≥85 cm in men and women, respectively, based on epidemiologic study findings (29).

## Prevalence of Sarcopenic Obesity

Accurate estimation of the prevalence of sarcopenic obesity is limited due to not only the lack of a universally adopted definition of sarcopenia but also the use of different body composition assessment techniques (30). In a 14-year prospective study of older adults (*n* = 4,652) more than 60 years of age by the National Health and Nutrition Examination Survey (NHANES) III, the prevalence rates of sarcopenic obesity were 18.1% in women and 42.9% in men (31). The study defined sarcopenia using the BIA-derived sex-specific cutoffs for ALM/ht^2^, as proposed by Janssen et al. (13) (men: ≤ 10.75 kg/m^2^ and women: ≤ 6.75 kg/m^2^). Obesity was based on the percentage of body fat (men: ≥27%, women: ≥38%). Previous studies in Korea have assessed the prevalence rates of sarcopenic obesity using information from the Korean NHANES (KNHANES) IV (32, 33) database, which measured skeletal muscle mass using DXA. The prevalence rate of sarcopenic obesity was 7.6% for men and 9.1% for women based on ALM/weight (%). However, the rate was nearly zero for men and women using the ALM/ht^2^ definition in the elderly population aged ≥65 years (32). Sarcopenic obesity was defined as class II sarcopenia with central obesity (WC: ≥90 cm for men and ≥85 cm for women) (29). Another study in Koreans (*n* = 2,221) aged over 60 years that used the same definition of sarcopenic obesity (ALM/weight (%): <2 SD from the reference values of young adult and central obesity) reported a prevalence rate of sarcopenic obesity of 6.1% for men and 7.3% for women (33). The rapidly increasing prevalence of obesity suggests the likely corresponding increase in sarcopenic obesity in these individuals.

## Health Consequences of Sarcopenic Obesity

### Disability and Institutionalization

Sarcopenic obesity is associated with disability. In a cohort study of 451 elderly men and women, subjects with sarcopenic obesity, defined according to ALM/ht^2^ and percent body fat, had a 2.5-fold increased risk of disability during an 8-year follow-up period than individuals without sarcopenic obesity (34); however, sarcopenia or obesity alone were not significantly associated with disability. The Concord Health and Aging Project reported that elderly men with sarcopenic obesity (defined based on ALM/BMI and % body fat according to the FNIH criteria) had a 2-fold higher risk of frailty and an ~1.5-fold increased risk of disability during the 7 years of follow-up (35). Sarcopenia was associated with poor functional outcomes while obesity alone was not associated with any adverse outcomes. However, several cross-sectional studies reported opposite or mixed results. Sarcopenia or sarcopenic obesity (low muscle mass and high % body fat) was not related to disability in people aged 70 years and older from the NHANES, although obesity was associated with an increased risk of functional limitation in both men and women (36). Another study reported that elderly women with sarcopenia only or with sarcopenic obesity did not have increased risks of disability, whereas those with obesity showed a 3-fold increased risk of disability (37).

Growing evidence indicates that muscle strength is a better indicator of aging-related functional decline than muscle mass. A meta-analysis of the relationship between body composition and muscle strength measures and functional decline in older men and women reported an association between dynapenia and obesity and long-term functional decline, respectively (20). In contrast, low muscle mass was not significantly associated with functional limitations. In the Invecchiare in Chianti (InCHIANTI, aging in the Chianti area) study, community-dwelling older adults with dynapenic obesity showed a steeper decline in walking speed and an increased risk of developing new mobility disability over the 6-year follow-up compared to those without obesity or dynapenia (38). A cross-sectional study from China found that dynapenic obesity (low handgrip strength and elevated BMI) was associated with increased risks of disability and slow gait speed compared to either dynapenia or obesity alone in an older Asian population (39). Similarly, a 2-year follow-up longitudinal study from Japan reported a higher risk of developing mobility limitations in older women with dynapenic obesity than in participants without dynapenia or obesity (40). Obesity alone was not associated with the incidence of mobility limitations. Data from the UK Biobank study recently showed that high BMI and low grip strength at baseline independently predicted lower physical activity levels as assessed by wrist-worn accelerometry at follow-up (41).

Physical performance, one of the components of sarcopenia, has predictive value for disability. Decreased physical performance, as assessed by gait speed, increased the risk of disability in elderly people (42). Another prospective cohort study of Health ABC participants showed increased risks of functional limitation and mortality in participants with slow gait speed (43). Furthermore, other tests of lower extremity function such as chair stand and standing balance showed comparable prognostic value for adverse health events.

Each component of sarcopenia has a different association with institutionalization. A large observational study using data from Health ABC participants found that low muscle mass was not independently associated with an increased risk of hospitalization (44). However, low muscle strength and poor physical performance were associated with increased risks of hospitalization (44). A Japanese population-based cohort study also reported that both low muscle strength and physical performance were risk factors for the certification of need of care (45). Moreover, physical performance measures such as gait speed have been associated with future hospitalization and institutionalization in a variety of populations (46–48). A recent prospective cohort study of patients with osteoporotic fractures found that none of the consensus definitions of sarcopenia nor the definition components of low muscle mass or low muscle strength was associated with increased risks of hospitalization or short-term nursing facility stay in older men and women. In contrast, the presence of slowness (based on gait speed) was associated with an increased likelihood of hospitalization (49, 50), although the association was attenuated after adjusting for confounding factors in men (49).

Few longitudinal studies have evaluated the relationship between sarcopenic obesity and institutionalization. Among elderly men in the Concord Health and Aging Project, low muscle mass and sarcopenic obesity (defined based on ALM/BMI and % body fat according to FNIH criteria) were not associated with institutionalization (35). However, the association between obesity and hospitalization or institutionalization is more obvious. For instance, participants with obesity (BMI ≥30 kg/m^2^) in the NHANES showed a higher likelihood of nursing facility use (51). A longitudinal observation study reported midlife obesity to be associated with an increased risk of nursing home admission in late life (52), an association that persists in older adults with obesity (53). A similar association was shown in a Japanese population-based cohort study that reported an increased risk of certification of need of care in older adults with obesity (BMI ≥27.5 kg/m^2^) (45).

### Mortality

Several prospective studies have investigated the relationship between sarcopenic obesity and the risk of mortality. However, the definitions of both sarcopenia and obesity vary across studies and the results have been conflicting. The British Regional Heart Study, a 6-year prospective study of 4,252 men aged 60–79 years, reported a 55% higher risk of mortality in men with a high WC (>102 cm) and low midarm muscle circumference (sarcopenic obesity) than in those without sarcopenia or obesity (54). Furthermore, in the extended 11-year follow-up of the British Regional Heart Study, men with sarcopenic obesity had a higher all-cause mortality risk [hazard ratio (HR): 1.72, 95% confidence interval (CI): 1.35–2.18] than those with sarcopenia without obesity (HR: 1.41, 95% CI: 1.22–1.63) and non-sarcopenic obese men (HR: 1.21, 95% CI: 1.03–1.42) (55). Another prospective study of 4,652 participants aged ≥60 years from the NHANES III with a 14-year follow-up showed higher risks of all-cause mortality in women with sarcopenia (HR: 1.35, 95% CI: 1.05–1.74) and sarcopenic obesity (based on ALM/ht^2^ and body fat measurement from BIA) (HR: 1.29, 95% CI: 1.03–1.60) than in women without sarcopenia or obesity (56). Meanwhile, women with obesity were not at a high risk of mortality, and no significant difference was observed in mortality risk in male participants with sarcopenia, obesity, and sarcopenic obesity. In contrast, the InCHIANTI study, which included 934 participants aged 65 years or older in their 6-year follow-up, reported no significant difference in mortality risks across six study groups according to calf skeletal muscle mass and BMI. However, individuals with sarcopenic obesity showed the lowest survival rate (57). Interestingly, low physical performance (measured using walking speed) was significantly associated with increased mortality in older adults (57). Recently, a long-term follow-up study of 2,309 Japanese-American elderly men from the Kuakini Honolulu Heart Program reported a significantly higher risk of mortality in the sarcopenia group (defined using ALM/ht^2^ with DXA) than in the non-sarcopenia non-obesity group using all three definitions of obesity (BMI, % body fat, and WC) (58). The risk of mortality was significantly higher in the sarcopenic obesity group defined using WC than in the non-sarcopenia non-obesity group (HR: 1.19, 95% CI: 1.02–1.38) but not in the groups defined using BMI and % body fat.

Measures of muscle strength, both knee extension and grip, were strong and independent predictors of mortality in older adults (7). The magnitude of association for both quadriceps and grip strength were similar (7). Although leg strength was more strongly associated with age itself than has grip strength (59, 60), grip strength is currently much easier to measure, thus has greater potential for incorporating into clinic practice. A growing body of evidence indicates that muscular strength, as measured using grip strength, is associated with a variety of health outcomes including mortality in older adults (61–65). Moreover, lower handgrip strength was correlated with increased risks of all-cause mortality (HR: 1.41, 95% CI: 1.30–1.52) and CVDs (HR: 1.63, 95% CI: 1.36–1.96) in a recent meta-analysis of 42 prospective cohort studies that included 3,002,203 participants (66). In a prospective study of 6,040 healthy men aged 45–68 years who were followed-up for over 30 years, adult men with the lowest tertile of handgrip strength at baseline had a higher risk for mortality, independent of BMI (67). Another prospective study from Taiwan reported a higher risk of mortality in participants with dynapenia without obesity than in those with obesity alone and even those with dynapenic obesity (defined using grip strength and WC) (68). Thus, dynapenia rather than obesity may have caused the unfavorable impact of dynapenic obesity. A similar observation was reported in a 33-year follow-up study that included 3,594 men and women aged 50–91 years from the Mini-Finland Health Examination Survey. In this study, among participants aged ≥70 years, the risk of mortality was higher in participants with dynapenic obesity (defined using grip strength and BMI) (HR: 1.23, 95% CI: 1.04–1.46) and dynapenia alone (HR: 1.30, 95% CI: 1.09–1.54) than in participants without dynapenia or obesity (69). Moreover, the English Longitudinal Study of Aging reported minimal differences in all-cause mortality between patients with dynapenic obesity (defined using grip strength and BMI) and those with dynapenia alone (70). In this study, weight loss combined with low muscle strength had the greatest risk of mortality. Likewise, recent studies have shown an association between overweight or obesity and a lower risk of CVD or CVD-associated death, whereas being underweight is associated with an increased risk of CVD, a phenomenon known as the obesity paradox (71, 72). Furthermore, several prospective studies reported a J-shaped association between BMI and both all-cause and cardiovascular mortality (73, 74). One possible explanation for the obesity paradox may be that overweight or obesity is associated with increased levels of lean mass (75). Older people with weight loss lose a greater percentage of lean mass than fat mass (76), which could contribute to the increased risk of CVD events after weight loss.

Slow walking speed in older people was associated with an increased risk of cardiovascular mortality in a cohort of 3,208 older men and women (77). Moreover, gait speed was associated with survival in pooled analysis of individual data from 9 cohort studies (pooled HR per 0.1 m/s: 0.88, 95% CI: 0.87–0.90, *P* < 0.001) (78). Short Physical Performance Battery (SPPB) score is a group of physical performance measures including gait speed, chair rises, and balance test, and it was associated with an increased risk of all-cause mortality in a meta-analysis (79). A comparative study among 3,099 older community-dwelling men and women revealed that slow gait speed and low SPPB scores were significant predictors for mortality, and the prognostic usefulness of SPPB and gait speed were similar, independently from gender (80). However, there is no study that evaluated the synergistic effects of low physical performance and obesity.

### Metabolic Diseases

Both sarcopenia and obesity are associated with metabolic disorders (81). Thus, sarcopenic obesity may have a greater impact on metabolic diseases and CVD-associated mortality than either sarcopenia or obesity alone (82–85). In a large cross-sectional analysis of 14,528 adults from the NHANES III, the sarcopenic obesity group (defined based on BIA-measured muscle mass and BMI) showed the highest risk of insulin resistance and dysglycemia (86). The Korean Sarcopenic Obesity Study (KSOS) cohort study showed that sarcopenic obesity [defined using DXA-measured ALM/weight (%) and visceral fat area] was associated with insulin resistance [Homeostatic Model Assessment of Insulin Resistance (HOMA-IR) score], inflammation (C-reactive protein level), and vitamin D deficiency (87). A cross-sectional study of 2,943 participants aged 60 years or older from KNHANES also reported that sarcopenic obesity [based on DXA-measured ALM/weight (%) and BMI] was associated with insulin resistance, metabolic syndrome, dyslipidemia, and vitamin D deficiency (88). Lim et al. observed a higher risk of metabolic syndrome among adults with sarcopenic obesity [defined using DXA-measured ALM/weight (%) and visceral fat area] [odds ratio (OR): 8.28, 95% CI: 4.45–15.40] than among those with obesity (OR: 5.51, 95% CI: 2.81–10.80) or sarcopenia (OR: 2.64, 95% CI: 1.08–6.44) in a cross-sectional study of 565 participants aged ≥65 years from the Korean Longitudinal Study on Health and Aging (89). In contrast, using the definition ALM/ht^2^, the ORs of metabolic syndrome were lower in the obesity group than in the sarcopenic obesity group (2.90 [95% CI: 1.28–6.57] vs. 4.80 [95% CI: 2.63–8.75]) (89). Similar findings were reported in a study of 600 community-dwelling older adults from Taiwan, in which sarcopenic obesity [defined based on BIA-measured ALM/weight (%) and BMI] was associated with the highest risk of metabolic syndrome (90). The sarcopenic obesity group had a higher risk of metabolic syndrome (OR: 11.59, 95% CI: 6.72–19.98) than the obesity group (OR: 7.53, 95% CI: 4.01–14.14) and sarcopenia group (OR: 1.98, 95% CI: 1.25–3.16). Our study comparing Australian and Korean populations observed a potential difference in the effects of low muscle mass on the risk of metabolic syndrome according to ethnicity (91).

Several cross-sectional studies of Korean populations of older adults from the KNHANES database reported sarcopenic obesity to be more strongly associated with increased risks of hypertension, dyslipidemia, and diabetes than sarcopenia or obesity alone (92–94). These studies defined sarcopenia as ALM/weight (%) since the association between sarcopenic obesity and cardiometabolic parameters was not definite when sarcopenia was indicated as ALM/ht^2^. The risk of hypertension was higher in the sarcopenia (OR: 2.48, 95% CI: 1.89–6.16), obesity (OR: 3.15, 95% CI: 2.76–3.59), and sarcopenic obesity (OR: 6.42, 95% CI: 4.85–8.48) groups than in the non-sarcopenia non-obesity group [defined using DXA-measured ALM/weight (%) and WC]. Furthermore, individuals with sarcopenic obesity [defined based on DXA-measured ALM/weight (%) and BMI] had a higher risk of dyslipidemia (OR: 2.82, 95% CI: 1.76–4.51) than men in the obesity (OR: 2.12, 95% CI: 1.11–4.07) and sarcopenia (OR: 1.46, 95% CI: 1.01–2.11) groups (92). Another Korean study reported a higher risk of diabetes in the sarcopenic obesity group [defined using DXA-measured ALM/weight (%) and WC] (OR: 2.16, 95% CI: 1.08–3.27) than in the sarcopenia group (OR: 1.24, 95% CI: 0.86–2.15) (93). Considering the association between sarcopenia and cardiometabolic risk factors, a cross-sectional study was performed to assess the association between sarcopenic obesity and the risk of CVD in Korean adults from the KNHANES database. The 10-year CVD risk of the participants was estimated using the Framingham risk score. The sarcopenic obesity group [based on the DXA-measured ALM/weight (%) and BMI] had a higher 10-year CVD risk than the non-sarcopenia non-obesity group (OR: 2.49, 95% CI: 1.53–4.06 in men; OR: 1.87; 95% CI: 1.02–3.41 in women) (94). In contrast, the risk of CVD was not high in the groups with sarcopenia or obesity alone.

However, studies on the relationship between sarcopenic obesity and CVD have reported contrasting results. A prospective cohort study of older men from the British Regional Heart Study did not observe increased risks of coronary heart disease (fatal or non-fatal myocardial infarction) or CVD (non-fatal myocardial infarction, non-fatal stroke, or fatal CVD) events in men with sarcopenic obesity (defined based on WC and midarm muscle circumference) (55). These men were at a higher risk of CVD-associated mortality. However, the risk was not significant in the adjusted model and was similar to that of individuals with sarcopenia or obesity alone. Moreover, several cross-sectional studies have reported that older adults with sarcopenic obesity [based on DXA-measured ALM/weight (%) and % body fat or BMI] did not have a significantly higher prevalence of CVD than adults without sarcopenia or obesity (34, 95–97).

Recent studies have investigated the relationship between low muscle strength and metabolic diseases. Relative handgrip strength, defined as handgrip strength normalized for BMI, was strongly negatively correlated with metabolic syndrome (98), hypertension (99), and dyslipidemia (100). Handgrip strength normalized by body weight was inversely associated with insulin resistance and type 2 diabetes (101). Furthermore, lower handgrip strength was associated with non-alcoholic fatty liver disease (102) and all-cause and CVD mortality (103). Some studies have reported an association between dynapenic obesity and metabolic disorders. A longitudinal study of 5,953 older adults from the English Longitudinal Study of Aging reported an association between dynapenic obesity (defined using grip strength and BMI) and an increased risk of type 2 diabetes (OR: 3.57, 95% CI: 2.04–6.24) (104). The Cardiovascular Health Study, a large prospective study of 3,366 community-dwelling older individuals, demonstrated an association between dynapenic obesity (based on handgrip strength and WC) and the highest risk of CVD (105). Compared to that in participants without sarcopenia or obesity, the risk of CVD did not significantly increase in the dynapenia or obesity groups. However, the risk of CVD increased by 23% in the dynapenia and obesity groups. In contrast, the risk of CVD was not significantly higher in the sarcopenic obesity group, when defined using BIA-measured muscle mass; thus, muscle strength may be more important predictor of CVD than muscle mass.

Chronic medical conditions such as hypertension, diabetes mellitus, and dyslipidemia were associated with lower walking speed and greater decline in walking speed in older people (106, 107). Moreover, in a longitudinal cohort study of 5,376 adults without CVD, higher cardiovascular risk scores at baseline were associated with future risk of poor physical performance at approximately 16 years of follow-up (108). All these results indicated that midlife cardiovascular risk factors likely contribute to poor physical function and disability in the elderly.

### Comorbidities

Low muscle mass and strength are associated with an increased fall risk in community-dwelling older adults. In elderly men, low muscle mass was associated with impaired balance and an increased risk of falls (109). A meta-analysis reported increased risks of any and recurrent falls in older adults with low muscle strength (110). Elderly individuals with sarcopenia as defined by the EWGSOP (low muscle mass plus either low muscle strength or low physical performance) were over three times more likely to fall during a 2-year follow-up period than individuals without sarcopenia (111). In contrast, a 5-year prospective cohort study that included 674 middle-aged and older adults reported an increased risk of falls in individuals with dynapenic obesity (based on trunk fat mass) but not in those with sarcopenic obesity (112). Consistently, dynapenic abdominal obesity (based on handgrip strength and WC) is associated with an increased risk of falls in older women; moreover, these relationships are stronger than those observed for obesity or dynapenia alone (113).

A cross-sectional study using data from 2,893 in KNHANES participants reported a high risk of knee osteoarthritis in the sarcopenic obesity [determined based on ALM/weight (%) and BMI] (OR: 3.51, 95% CI: 2.15–5.75) and non-sarcopenic obesity (OR: 2.38, 95% CI: 1.80–3.15) groups but not in the sarcopenic non-obesity group (114). The risk of osteoporosis was higher in the sarcopenic obesity group (based on ALM/ht^2^ and % body fat) (OR: 8.67, 95% CI: 4.19–17.94 in men and OR: 2.93, 95% CI: 1.99–4.32 in women) (115). Similarly, another study that defined sarcopenic obesity as low muscle mass or muscle strength, with obesity defined according to fat mass, reported lower bone mineral density and a higher risk of non-vertebral fracture in older adults with sarcopenic obesity than in adults without sarcopenia, without obesity, and only obesity (116).

Several reports have shown the relationship between sarcopenic obesity and psychological health problems. A 6-year prospective study of 3,862 participants from the English Longitudinal Study of Aging reported an increased risk of depression in patients with sarcopenic obesity (defined based on grip strength and BMI) (OR: 1.79, 95% CI: 1.10–2.89) than in individuals without sarcopenia or obesity. The risk of depression was not significantly higher in participants with obesity or sarcopenia alone (117). Another study reported similar findings, showing that sarcopenic obesity—defined based on low muscle mass plus either low muscle strength or low physical performance, with obesity defined according to % body fat—was positively associated with depressive symptoms compared with non-sarcopenic non-obesity (118). Sarcopenia or obesity alone were not associated with depressive symptoms.

## Discussion

Sarcopenic obesity is a global health phenomenon due to both the rapid increase in the number of elderly individuals and the obesity epidemic. Aging-associated increases in visceral fat and reduced muscle mass are correlated with multiple adverse cardiometabolic effects and contribute to poor health outcomes (119). Several biological pathways lead to age-related sarcopenic obesity. Aging lowers the resting metabolic rate and metabolic adaptation including adaptive thermogenesis, perpetuating low muscle mass, and increased body fat (120, 121). Reduced resting metabolic rate, physical activity, mitochondrial volume, and oxidative capacity with age contribute to aging-related decreases in muscle mass and strength (122–124). Moreover, the aging-related changes in body fat distribution include a loss of subcutaneous fat, accumulation of visceral fat, and ectopic fat deposition (125). Sex-specific hormonal changes are an important factor related to sarcopenic obesity. In women, declining estrogen levels after menopause result in increased body weight and fat mass as well as shifts in the accumulation of fat from subcutaneous to visceral deposits (126, 127). In older men, total testosterone levels decline by about 1% per year of age, with lower levels associated with sarcopenia, lower muscle strength, poorer physical performance, and increased fall risk (128–130). Randomized controlled trials of various formulations of testosterone supplementation in older men have reported improvements in body composition including increased lean body mass and reduced fat mass (131, 132). However, the effects of testosterone therapy on muscle strength or physical performance are conflicting. Increasing serum testosterone levels to low- to mid-normal ranges through the administration of testosterone for several months in healthy older men resulted in increased muscle strength (133–136) and improved physical performance (134, 135). In contrast, a randomized controlled trial of testosterone replacement for 6 months in healthy older men reported increased lean body mass and decreased fat mass, with no increase in functional mobility or muscle strength (137). Another study on testosterone supplementation for 1 year did not observe improved walking distance in older men with reduced testosterone levels (138).

**Figure 1:**
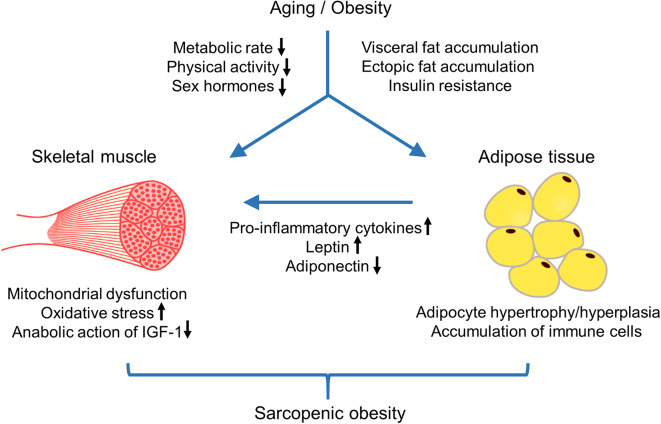
Schematic biological pathways that lead to sarcopenic obesity with age. Aging accompanies with reduced metabolic rate, decreased physical activity, and sex-specific hormonal changes. These changes contribute to aging-related decrease in muscle mass and strength as well as increase in body fat and unfavorable changes in body fat composition, such as a loss of subcutaneous fat, an accumulation of visceral fat, and ectopic fat deposition. Obesity in aged individuals stimulates ectopic fat accumulation in skeletal muscles, which leads to marked impairment of mitochondria fatty acid oxidation, increased oxidative stress, and insulin resistance, resulting in decline in muscle mass and strength. Moreover, obesity-associated adipose tissue inflammation can affect skeletal muscle mass and function by the increased production of pro-inflammatory cytokines and adipokine leptin, and decreased action of adiponectin and IGF-1. Aging-associated sarcopenic obesity is correlated to multiple adverse cardiometabolic effects and contributes to poor health outcomes.

Obesity in aged individuals stimulates sarcopenia by altering skeletal muscle lipid metabolism, insulin resistance, and inflammatory pathways (139–141). Obesity promotes the deposition of ectopic fat into skeletal muscle, which can negatively affect sarcopenia (142, 143). Both intermuscular adipose tissue and intramuscular lipid metabolites can lead to marked impairment in mitochondrial fatty acid oxidation, increased lipolysis, and increased oxidative stress (141, 144, 145). These events can promote lipotoxicity, insulin resistance, and inflammation in skeletal muscle, resulting in decreased muscle mass and muscle fiber contractility (141, 146, 147). Obesity-associated adipose tissue inflammation is another mechanism leading to sarcopenia (141). Obesity promotes inflammatory pathways in visceral fat through adipocyte hypertrophy and hyperplasia and the accumulation of immune cells including macrophages, mast cells, and T lymphocytes producing pro-inflammatory cytokines (148–150). Moreover, increased secretion of leptin from adipose tissue upregulates the production of pro-inflammatory cytokines such as tumor necrosis factor (TNF)α and interleukin (IL)-1β (151). Elevated TNFα directly impairs adiponectin signaling, thus inhibiting mitochondrial biogenesis and myogenesis in human skeletal muscle cells (152). Pro-inflammatory cytokines can also negatively affect the anabolic action of insulin-like growth factor 1 (IGF1), resulting in an increased incidence of frailty in older men along with the age-related reduction in testosterone levels (153). All these changes can induce and/or exacerbate decreased skeletal muscle mass and function. The mechanisms of developing sarcopenic obesity with age was schematically depicted in Figure 1.

As sarcopenia and obesity share pathological factors including aging, hormones, and immunological factors, sarcopenic obesity affects the risks of adverse health outcomes more than sarcopenia or obesity individually. A growing body of evidence has shown the associations of sarcopenic obesity with increased risks of disability, institutionalization, mortality, metabolic diseases, CVD, and other comorbidities, compared to sarcopenia or obesity alone. However, the findings of studies on the relationship between sarcopenic obesity and cardiometabolic risk are discordant. The inconsistencies in the associations between sarcopenic obesity and health outcomes may be attributed to differences in sample sizes, study population characteristics, and heterogeneity in the definitions of sarcopenic obesity. Furthermore, the role of dynapenia, a condition characterized by an age-related decline in muscle strength, is now considered a principal determinant of sarcopenia, overtaking the role of low muscle mass (20, 21). Notably, the EWGSOP2 reported low muscle strength as a key characteristic of sarcopenia to facilitate the prompt identification of sarcopenia in practice (19). In this revised consensus, low physical performance is proposed as an indicator of severe sarcopenia. Physical performance involves not only muscles but also bones, balance, and cardiovascular capacity, beyond muscle function measures (154). Further studies to elucidate the molecular mechanisms of age-associated changes in muscle mass, muscle strength, and physical performance are needed to prevent and treat sarcopenia; these biological pathways should be considered along with age-associated obesity.

## Author Contributions

ER contributed to the literature search and original draft preparation. KC contributed to the study conceptualization and text review and editing. All authors have read and agreed to the published version of the manuscript.

## Conflict of Interest

The authors declare that the research was conducted in the absence of any commercial or financial relationships that could be construed as a potential conflict of interest.
